# Prediction models and risk scores in different types of heart failure: a review

**DOI:** 10.3389/fmed.2025.1652307

**Published:** 2025-11-21

**Authors:** Yue Wei, Siyu Liu, Yunying Mu, Xiaoyu Liang, Ziyi Chen, Yongcheng Liu, Guoju Dong

**Affiliations:** 1 Department of Cardiovascular Internal Medicine, Xiyuan Hospital, China Academy of Chinese Medical Sciences, Beijing, China; 2 National Resource Center for Chinese Materia Medica, China Academy of Chinese Medical Sciences, Beijing, China; 3 National Clinical Research Center for Chinese Medicine Cardiology, Xiyuan Hospital, China Academy of Chinese Medical Sciences, Beijing, China

**Keywords:** heart failure, heart failure with mildly reduced ejection fraction, heart failure with preserved ejection fraction, heart failure with reduced ejection fraction, prediction model

## Abstract

**Background:**

Heart failure (HF) is a leading cause of global disease burden and mortality. Accurate prognosis assessment is critical for reducing the risk of adverse events. In recent years, numerous predictive models have been developed for different HF subtypes. However, the quality of existing models varies considerably, and there remains a lack of consensus on models suitable for widespread clinical application. This study systematically reviews the current landscape of HF prediction models, analyzes their strengths and limitations, and provides guidance for future research.

**Methods:**

This review systematically retrieved studies on prognostic prediction models for HF from databases including PubMed and Embase, with a search period spanning from the inception of each database to 19 September 2025. The risk of bias of the included studies was assessed using the prediction model risk of bias assessment tool, and the performance of the prediction models was evaluated through metrics such as the C-index and calibration.

**Results:**

A total of 46 prediction models from 38 studies were included. According to target population classification, 14 models were developed for predicting outcomes in HF patients with reduced ejection fraction, nine models were applicable to HF patients with preserved ejection fraction, one model targeted HF patients with mildly reduced ejection fraction, and the remaining 22 were designed for all HF patients regardless of subtype. The risk of bias assessment showed that 10 models had a high risk of bias, 21 models demonstrated an unclear risk of bias, and 15 models exhibited a low risk of bias. The study systematically summarized each model's study cohort, modeling methodology, predictors, outcomes, prediction performance, presentation format, as well as strengths and limitations.

**Conclusion:**

Refining the methodological processes of model construction—including optimizing study cohort selection, updating predictor screening (such as incorporating novel biomarkers, imaging indicators, and multi-omics data), improving modeling strategies, and enhancing model presentation—will contribute to the development of more accurate and clinically applicable prediction models. Such advancements hold significant potential for improving clinical outcomes in patients across all types of HF. This review provides a substantive reference for future research in this field.

## Introduction

Heart failure (HF) represents one of the major global public health challenges. According to the Global Burden of Disease (GBD) study, approximately 64.3 million individuals worldwide suffer from HF, with the disease burden continuing to escalate (1). Despite advances in therapeutic strategies, patient prognosis remains suboptimal, with annual mortality rates in high-risk patients ranging from 15 to 30% and 5-year mortality rates reaching 50%−75% (1, 2). Readmission rates also persist at elevated levels (1, 2).

Early risk assessment is crucial for improving patient outcomes and optimizing healthcare resource allocation. High-risk patients require enhanced monitoring and intervention, with consideration given to ventricular assist device implantation or heart transplantation where necessary; extremely high-risk patients should focus on symptom relief and end-of-life care; while low-risk patients may undergo appropriately simplified follow-up to conserve healthcare resources (3, 4). Notably, the 2021 European Society of Cardiology (ESC) guidelines classify HF into three subtypes based on left ventricular ejection fraction (LVEF): HF with reduced ejection fraction (HFrEF), HF with mildly reduced ejection fraction (HFmrEF), and HF with preserved ejection fraction (HFpEF), advancing HF management into a stage of precision medicine (5). The baseline characteristics, incidence, and prognosis of different types of HF differ significantly. Consequently, prognostic prediction models tailored to distinct HF subtypes have emerged as a research priority in recent years (6).

However, existing prediction models exhibit substantial heterogeneity in predictive performance, generalizability, and clinical utility, necessitating systematic evaluation and comparison. This study undertakes a review of prediction models for different HF subtypes, summarizing their strengths and limitations to provide direction for future research, thereby advancing the implementation of precision medicine in HF management.

## Methods

### Search strategy

Studies on HF prediction models were systematically searched in PubMed and Embase from inception to September 19, 2025. Search terms included “heart failure,” “heart failure with preserved ejection fraction,” “heart failure with mildly reduced ejection fraction,” “heart failure with reduced ejection fraction,” “prognosis,” “prediction,” “risk score,” and their synonyms. The detailed search strategies were provided in Tables 1, 2. Additionally, the reference lists of relevant literature were screened to avoid omissions (7).

**Table 1:** Search terms and search strategy with full results (Database: PubMed).

**Searches**	**Results**
(“Heart Failure”[Mesh] OR “heart failure”[tiab] OR “HFrEF”[tiab] OR “HFpEF”[tiab] OR “HFmrEF”[tiab] OR HF[tiab]) AND (“Prognosis”[Mesh] OR “Predictive Value of Tests”[Mesh] OR “Risk Assessment”[Mesh] OR “Models, Statistical”[Mesh] OR “Nomograms”[Mesh]) AND (“prediction model”[tiab] OR “predictive model”[tiab] OR “risk model”[tiab] OR “risk score”[tiab] OR “prognostic model”[tiab] OR “prognostic score”[tiab] OR “risk prediction”[tiab] OR “risk stratification”[tiab])	5,351

**Table 2:** Search terms and search strategy with full results (Database: Embase).

**No**.	**Query**	**Results**
#16	#4 AND #10 AND #15	14,758
#15	#11 OR #12 OR #13 OR #14	284,764
#14	‘risk prediction':ab,ti OR ‘risk stratification':ab,ti	128,186
#13	risk score':ab,ti OR ‘prognostic score':ab,ti OR ‘prediction score':ab,ti	68,914
#12	prediction model':ab,ti OR ‘predictive model':ab,ti OR ‘risk model':ab,ti OR ‘prognostic model':ab,ti	98,906
#11	‘predictive model'/exp	37,665
#10	#5 OR #6 OR #7 OR #8 OR #9	2,802,699
#9	‘nomogram'/exp	37,302
#8	‘statistical model'/exp	802,750
#7	‘risk assessment'/exp	873,787
#6	‘predictive value'/exp	306,609
#5	‘prognosis'/exp	1,031,324
#4	#1 OR #2 OR #3	895,066
#3	hfref:ab,ti OR hfpef:ab,ti OR hfmref:ab,ti OR ‘heart decompensation':ab,ti OR ‘heart failure with reduced ejection fraction':ab,ti OR ‘heart failure with preserved ejection fraction':ab,ti OR ‘heart failure with mid range ejection fraction':ab,ti	30,771
#2	heart failure':ab,ti OR hf:ab,ti OR ‘cardiac failure':ab,ti OR ‘heart decompensation':ab,ti	480,979
#1	‘heart failure'/exp	786,144

### Inclusion and exclusion criteria

Studies on multivariable prediction models for predicting the prognosis of HF in adults (age ≥18 years) were included. “Prognosis” was defined as the occurrence of major long-term endpoints, such as all-cause mortality, all-cause hospitalization/rehospitalization, and cardiovascular/cerebrovascular events. The exclusion criteria for the study were as follows: (1) studies of diagnostic models predicting the occurrence of HF (rather than its prognosis); (2) studies that only validated existing models without developing new ones; (3) studies that merely explored the association between risk predictors and HF prognosis without constructing a complete prediction model; (4) studies restricted to specific subgroups of HF patients (such as those with specific comorbidities or caused by specific etiologies); (5) reviews, systematic reviews, meta-analyses, or similar studies; (6) other studies unrelated to this research topic; (7) studies of predictive models lacking external validation (a model developed without external validation could still be considered externally validated if it was validated independently by other studies).

Literature screening and management were conducted using the Covidence platform and EndNote 21. Two investigators (Yue Wei, Siyu Liu) independently screened studies for eligibility based on titles, abstracts, and full texts (including Supplementary materials). Any discrepancies were resolved through discussion or by arbitration from a third investigator (Yunying Mu) to reach consensus.

### Data extraction and risk of bias assessment

Two investigators (Yue Wei, Siyu Liu) independently extracted information from each eligible study using a standardized data extraction sheet. We extracted the following information: first author name, publication date, participant cohort and regions, number of participants, methods, outcomes, predictors, predictive performance [Concordance statistic (C-statistic), validation, calibration, decision curve analysis (DCA), net reclassification improvement (NRI)], and model presentation.

Two investigators (Yue Wei, Siyu Liu) assessed the risk of bias for each eligible model using the Prediction Model Bias Risk Assessment Tool (PROBAST) (8). Any discrepancies were resolved through discussion and consensus with a third investigator (Yunying Mu).

## Results

### Study selection

**Figure 1:**
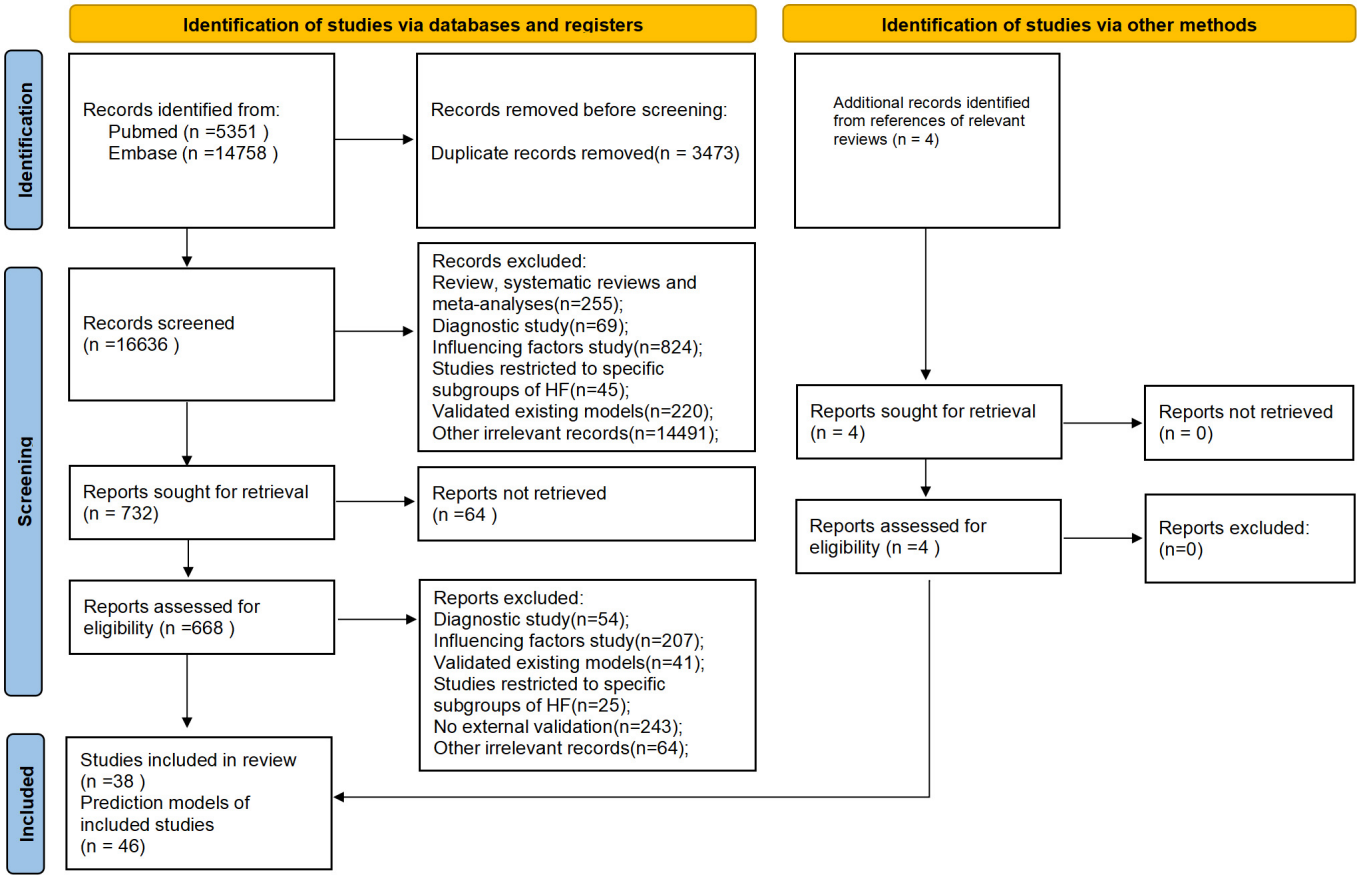
PRISMA flowchart of the literature screening process.

A total of 20,113 studies were initially identified. After removing 3,473 duplicates, 38 studies comprising 46 prediction models met the eligibility criteria following a review of titles, abstracts, and full-text articles (Figure 1). Notably, five of these studies reported on two or more prediction models. Among the included prediction models, 14 models from nine studies (9–17) were developed to predict outcomes in HFrEF patients, nine models from eight studies focused on HFpEF patients (18–25), and one model from a single study (26) targeted HFmrEF patients. The remaining 22 prediction models, derived from 20 studies (27–46), were designed for all HF patients regardless of subtype.

### Characteristics of included prediction models

As shown in Tables 3–5, among the 46 included prediction models, 31 (67%) were developed based on published cohort studies, while the remaining 15 (33%) utilized data from outpatient or inpatient recruitment, original electronic health records (EHR), or registry data. Only 11 (24%) prediction models were derived from study populations with broad geographical representation, covering most regions globally; the populations of the remaining prediction models were primarily concentrated in North America and Europe, with only 4 models specifically developed for Asian populations.

**Table 3:** Prediction models for HFrEF.

**Prediction model, year**	**Training group**	**Validation group**	**Participant cohort**	**Regions**	**Method**	**Predictors**	**Outcome**	**C-statistics**	**Validation**	**Calibration**	**Model presentation**
HFSS (9), 1997	268	199	Ambulatory patients (OS)	America	Cox proportional hazards analysis	Ischemic cardiomyopathy, sodium, peak Vo2, pulmonary capillary wedge pressure, intraventricular conduction delay, LVEF, HR, BP	Urgent transplant or death without transplant in 1 year	0.74 ± 0.05	Temporal validation + geographic validation	NA	Risk score
OPTIME-CHF (10) , 2004	949	NA	OPTIME-CHF trial (IS)	America	Cox proportional hazards analysis	Age, sodium, NYHA Class, SBP, BUN	60-day mortality	0.76	Bootstrap + geographic validation (93)	Calibration plot	Nomogram
SHFM (11), 2006	1,125	9,942	PRAISE1 study (IS);ELITE2 study (IS); Val-HeFT study (IS); UW study (OS); RENAISSANCE study (IS); IN-CHF study (OS)	Almost all major geographic regions	Cox proportional hazards analysis	Hemoglobin, lymphocytes, UA, total cholesterol, sodium, LVEF, NYHA Class, weight, SBP, ACEI, β-blocker, ARB, statin, allopurinol, aldosterone blocker, diuretics, ICD	1-year survival	0.729 (95% CI: 0.714–0.744)	Geographic validation	Calibration plot	Web-based calculator
ESCAPE (12), 2010	433	471	ESCAPE trial (IS); FIRST Trial (IS)	America and Canada	Cox proportional hazards analysis	BNP, cardiopulmonary resuscitation or mechanical ventilation during hospitalization, BUN, sodium, age, diuretic, β-blocker, 6-min walk	6-month mortality	0.78 (95% CI: 0.68–0.83)	Bootstrap + geographic validation	Calibration plot	Risk score
SHOCKED[13) , 2012	17,991	27,893	ICD registry cohort (OS)	America	Cox proportional hazards analysis	CKD, age, COPD, diabetes, NYHA, AF, LVEF	All-cause mortality within 4 years of primary prevention ICD implantation	0.74 (95% CI: 0.74–0.75)	Temporal validation + geographic validation	HL test *p*-value: 0.096	Nomogram
BCN-Bio-HF (14), 2014	864	NA	Outpatient clinics (OS)	Spain	Cox proportional hazards analysis	Age, sex, NYHA class, LVEF, sodium, eGFR, hemoglobin, diuretic, β-blocker, ACEI, ARB, statin, hs-cTnT, ST2, NT-proBNP	1/2/3-year mortality	0.793 (95% CI: 0.776–0.817)	Bootstrap + Geographic validation (94)	HL test *p*-value: 0.37/0.49/0.46	Web-based calculator
BIOSTAT-CHF risk score (15), 2017	2,516	1,738	BIOSTAT-CHF cohort (IS)	121 European countries	Cox proportional hazards analysis	Age, BUN, NT-proBNP, hemoglobin, β-blocker; age, previous hospitalization owing to HF, oedema, SBP, eGFR; age, previous hospitalization owing to HF, edema, NT-proBNP, SBP, hemoglobin, high-density lipoprotein, sodium, β-blocker	All-cause mortality; first HFH; the composite outcome of all-cause mortality and HFH	0.73/0.68/0.70	Bootstrap + geographic validation	Calibration plot	Web-based calculator
PREDICT-HF (16) , 2020	8,399	7,016 18,968	PARADIGM-HF trial (IS); ATMOSPHERE trial (IS); SwedeHF study (OS)	Almost all major geographic regions	Cox proportional hazards analysis	Age, sex, race, region, duration of HF, NYHA class, LVEF, diabetes, MI, peripheral arterial disease, β-blocker, ARNI, bundle-branch block, PCI, SBP, bilirubin, UA, albumin, potassium, hemoglobin, total cholesterol, NT-proBNP, BNP Age, sex, race, region, duration of HF, NYHA class, LVEF, diabetes, MI, peripheral arterial disease, β-blocker, ARNI, PCI, BMI, SBP, potassium, albumin, UA, bilirubin, absolute neutrophil count, hemoglobin, low-density lipoprotein, BNI, NT-proBNP, BNP Sex, race, region, duration of HF, NYHA class, LVEF, diabetes, MI, peripheral arterial disease, β-blocker, ARNI, prior HFH, valvular heart disease, bundle-branch block, bilirubin, UA, albumin, potassium, absolute neutrophils count, hemoglobin, low density lipoprotein, BUN, absolute lymphocytes count, NT-proBNP, BNP	CV death/all-cause mortality/composite of CV death or HFH in both 1 and 2 years	0.73 (95%CI: 0.71–0.75)/0.71 (95% CI: 0.69–0.73); 0.71 (95%CI: 0.69–0.74)/0.70 (95% CI: 0.67–0.72); 0.74 (95%CI: 0.71–0.76)/0.71 (95% CI: 0.70–0.73)	Temporal validation + geographic validation	Calibration plot	Risk score
PARADIGM-HFrEF (17) , 2021	7,156	5,968	PARADIGM-HF trial (IS); ATMOSPHERE trial (IS)	Almost all major geographic regions	Fine-Gray regression	NYHA class, NT-proBNP, sex, race, CABG or PCI, cancer, MI, ARNI/ACEI, QRS duration, ECG left ventricular hypertrophy; NYHA class, NT-proBNP, LVEF, ischemic etiology, SBP, HF duration, ECG bundle branch block, albumin, chloride, creatinine	Sudden death; pump failure death	0.66 (95% CI: 0.64–0.69); 0.75 (95% CI: 0.72–0.78)	Bootstrap+geographic validation	Calibration plot	Risk score

NA, not applicable; IS, interventional study; OS, observational study; HL, Hosmer–Lemeshow; LVEF, left ventricular ejection fraction; HR, heart rate; BP, blood pressure; OPTIME-CHF, Outcomes of a Prospective Trial of Intravenous Milrinone for Exacerbations of Chronic Heart Failure; NYHA, New York Heart Association; SBP, systolic blood pressure; BUN, blood urea nitrogen; PRAISE1,the Prospective Randomized Amlodipine Survival Evaluation;ELITE2, Evaluation of Losartan in the Elderly; Val-HeFT, Valsartan Heart Failure Trial; UW,University of Washington; RENAISSANCE, Randomized Enbrel North American Strategy to Study Antagonism of Cytokines; IN-CHF, Italian Heart Failure Registry; UA, uric acid; ACEI, angiotensin-converting enzyme inhibitor; ARB, angiotensin II receptor blocker; ICD, implantable cardioverter defibrillator; ESCAPE, Evaluation Study of Congestive Heart Failure and Pulmonary Artery Catheterization Effectiveness; FIRST, Flolan International Randomized Survival Trial; CKD, chronic kidney disease; eGFR, estimated glomerular filtration rate; hs-cTnT, high-sensitivity cardiac troponin T; ST2, soluble toll-like receptor-2; NT-proBNP, N-terminal pro-brain natriuretic peptide; PARADIGM-HF, Prospective Comparison of ARNI With ACEI to Determine Impact on Global Mortality and Morbidity in Heart Failure; ATMOSPHERE, Aliskiren Trial to Minimize Outcomes in Patients; CABG, coronary artery bypass grafting; PCI, percutaneous coronary intervention; MI, myocardial infarction; ARNI, angiotensin receptor–neprilysin inhibitor; ECG, Electrocardiogram; BIOSTAT-CHF, Biology Study to Tailored Treatment in Chronic Heart Failure; HFH, heart failure hospitalization; SwedeHF, Swedish Heart Failure Registry; MI, myocardial infarction; CV, cardiovascular.

**Table 4:** Prediction models for HFpEF and HFmrEF.

**Prediction model, year**	**Training group**	**Validation group**	**Participants cohort**	**Regions**	**Method**	**Predictors**	**Outcome**	**C-statistics**	**Validation**	**Calibration**	**Model presentation**
ARIC-HFpEF (18), 2017	1,852	821	ARIC study (OS);	America	Logistic regression	Age, race, cerebrovascular disease, COPD, AF/atrial flutter, sodium, BUN, hemoglobin, BNP, SBP, HR, hypoxia, BMI	All-cause mortality within 28 days and within 1 year	0.73, 0.71	Temporal validation	HL test *p*-value: 0.51/0.29	Web-based calculator
3A3B (19), 2019	1,277	835 170	CHART-2 study (OS); TOPCAT study (OS); ASIAN-HF study (OS)	America;Asia	Cox proportional hazards analysis	Age, anemia, albumin, BUN, BMI, BNP (or NT-proBNP)	5-year all-cause mortality	0.708	Bootstrap+geographic validation	Calibration plot	Risk score
MEDIA-ECHO score (20), 2021	515	286	MEDIA study (OS); KaRen study (OS)	Europe; Swedish; French	Cox proportional hazards analysis	Age, eGFR, AF, HF status, PAPS, inferior vena cava collapsibility, E/e', lateral s', NT-proBNP	Composite of admission for worsening HF or cardiovascular causes and cardiac death	0.775 (95% CI: 0.727–0.824)	Geographic validation (95)	NA	Risk score
I-PRESERVE (21), 2021	4,116	2,556 3,401	I-PRESERVE study (IS); CHARM study (IS); TOPCAT study (OS)	Countries across all major geographic regions	Fine-Gray regression	Age, sex, LVEF, HR, diabetes, MI, HFH within previous 6 months; age, sex, LVEF, DBP, HR, diabetes, AF, dyslipidemia	Sudden death; pump failure death;	0.71 (95% CI: 0.68–0.75); 0.78 (95% CI: 0.75–0.82)	Geographic validation	NA	Risk score
EMPEROR-Preserved (22) , 2022	5,988	1,251	EMPEROR-Preserved (IS); the PARAGON-HF study (IS)	Countries across all major geographic regions	Cox proportional hazards analysis	NT-proBNP, hs-cTnT, time since last hospitalization, NYHA class, COPD, insulin-treated diabetes, hemoglobin, time since HF diagnosis	Composite outcome of HF hospitalization or cardiovascular death	0.748 (95% CI: 0.732–0.764)	Temporal validation+geographic validation	HL test *p*-value:0.198	Web-based calculator
PREDICT-HFpEF (23), 2024	6,263	4,796 4,128	DELIVER study (IS); PARAGON-HF study (IS); I-PRESERVE study (IS);	Countries across all major geographic regions	Cox proportional hazards analysis	NT-proBNP, HFH within the past 6 months, creatinine, diabetes, geographic region, HF duration, SGLT2i, COPD, transient ischemic attack/stroke, any previous HFH, HR	1-year and 2-year the composite of HFH or CV death	0.73 (95%CI: 0.71–0.75)/0.71 (95% CI: 0.70–0.73);	Geographic validation	Calibration plot	Web-based calculator
MODEL 1 (24) , 2025	6,935	2,484	Hospitalized patients (OS);	China;	Cox proportional hazards analysis+LASSO regression	NT-proBNP, albumin, age, NYHA class, C-reactive protein, right atrial end-systolic diameter, hemoglobin, COPD, hyponatemia, PCI	3-year all-cause mortality	0.764 (95% CI: 0.742–0.786)	Bootstrap+temporal validation	HL test *p*-value: 0.12	Nomogram
MODEL2 (25) , 2025	766	803	Hospitalized patients (OS)	China	ML	Age, NYHA class, MI, AF, MLR, BNP, LVEF, E/e', ACEI/ARB/ARNI	Readmission due to cardiovascular reasons within 1 year	0.87 (95 %CI: 0.83–0.91)	Temporal validation	Calibration plot	Web-based dynamic nomogram
APSELNH (26) , 2024	790	338	Hospitalized patients (OS)	China;	Cox proportional hazards analysis+LASSO regression+RSF	ACEI/ARB/ARNI, PCI/CABG, stroke, eGFR, NT-proBNP, NYHA class, healthcare	All-cause mortality in 1,3,5 years	0.834 0.844 0.865	Bootstrap+geographic validation	Calibration plot	Web-based calculator

NA, not applicable; IS, interventional study; OS, observational study; HL, Hosmer–Lemeshow; ARIC, Atherosclerosis Risk in Communities; COPD, chronic obstructive pulmonary disease; AF, atrial fibrillation; BUN, blood urea nitrogen; BNP, brain natriuretic peptide; SBP, systolic blood pressure; HR, heart rate; BMI, body mass index; CHART-2, Chronic Heart Failure Registry and Analysis in the Tohoku District-2; TOPCAT, Treatment of Preserved Cardiac Function Heart Failure with an Aldosterone Antagonist; ASIAN-HF, Asian Sudden Cardiac Death in Heart Failure; NT-proBNP, N-terminal pro-brain natriuretic peptide; MEDIA, European MEtabolic Road to DIAstolic Heart Failure; eGFR, estimated glomerular filtration rate; HF, heart failure; PAPS, pulmonary arterial pressure, systolic; E, early diastolic mitral inflow velocity; e', early diastolic mitral annular tissue velocity; I-PRESERVE, Irbesartan in Heart Failure With Preserved Ejection Fraction Study; CHARM, Candesartan in Heart Failure, A Randomized Multinational Strategy Trial; LVEF, left ventricular ejection fraction; MI, myocardial infarction; HFH, heart failure hospitalization; DBP, diastolic blood pressure; EMPEROR-Preserved, Empagliflozin Outcome Trial in Patients with Chronic Heart Failure and a Preserved Ejection Fraction; PARAGON-HF, Angiotensin Receptor Neprilysin Inhibition in Heart Failure With Preserved Ejection Fraction; hs-cTnT, high-sensitivity cardiac troponin T; NYHA, New York Heart Association; DELIVER, Dapagliflozin Evaluation to Improve the Lives of Patients With Preserved Ejection Fraction Heart Failure; SGLT2i, sodium–glucose cotransporter 2 inhibitor; LASSO, least absolute shrinkage and selection operator; PCI, percutaneous coronary intervention; ML, machine learning; MLR, monocyte-to-lymphocyte ratio; ACEI, angiotensin-converting enzyme inhibitor; ARB, angiotensin II receptor blocker; ARNI, angiotensin receptor–neprilysin inhibitor; RSF, random survival forest; CABG, coronary artery bypass grafting.

**Table 5:** Prediction models for all HF regardless of types.

**Prediction model, year**	**Training group**	**Validation group**	**Participants cohort**	**Regions**	**Method**	**Predictors**	**Outcome**	**C-statistics**	**Validation**	**Calibration**	**Model presentation**
EFFECT-HF (27), 2003	2,624	1,407	CBC(OS); the EFFECT study(OS)	Canada	Logistic regression	Age, RR, SBP, BUN, cerebrovascular disease, dementia, COPD, hepatic cirrhosis, cancer, hemoglobin	All-cause mortality in 30-day and 1-year	0.79/0.76	Bootstrap+ temporal validation	HL test *p*-value > 0.05	Risk score
ADHERE (28) , 2005	33,046	32,229	Hospitalized patients (OS);	America	Logistic regression+ CART	BUN, SBP, creatinine	In-hospital mortality	0.757	Temporal validation	HL test *p*-value: 0.67	Web-based calculator
CHARM (29), 2006	7,599	—	CHARM study(IS)	26 countries in Europe, America, Canada, South Africa and Australia;	Cox proportional hazards analysis	Age, LVEF, diabetes, BMI, NYHA class, current smoker, bundle branch block, cardiomegaly, prior HFH, DBP, diagnosis of CHF over 2 years ago, MI, dependent oedema, HR, pulmonary crackles, pulmonary edema, mitral regurgitation, AF, rest dyspnea, candesartan	2-year combined endpoint of CV death or hospitalization for the worsening HF/all-cause mortality	0.75	Bootstrap+ geographic validation (96)	Calibration plot	Risk score
OPTIMIZE-HF (30) , 2008	48,612	937 181,830	OPTIME-CHF trial(IS); ADHERE study (IS)	America	Cox proportional hazards model, logistic regression	Age, HR, SBP, sodium, Cr, primary cause of admission, left ventricular systolic dysfunction	60- to 90-day post-discharge mortality	0.753	Bootstrap+ geographic validation	Calibration plot	Nomogram
GWTG-HF (Peterson) (31), 2010	27,850	11,333	GWTG-HF program (IS)	America	Logistic regression analysis	Age, SBP, BUN, HR, sodium, COPD, race	In-hospital mortality	0.75	Split-sample+ geographic validation (97)	HL test *p*-value: 0.604	Risk score
PROTECT 7-day (32) , 2012	2,033	1,435	PROTECT trial (IS); VERITAS trial (IS)	North America, Europe, Israel, Argentina	Cox proportional hazards analysis	BUN, albumin, SBP, HR, RR, cholesterol, hospitalization for HF in the past year, diabetes	7-day composite endpoint of death, HFH or worsening HF	0.67	Bootstrap+ geographic validation	HL test *p*-value: 0.67	Risk score
EHMRG (33) , 2012	7,433	5,158	Patients presenting to ED with ADHF(OS)	Canada	Logistic regression	Age, transported by EMS, oxygen saturation, HR, SBP, potassium, Cr, troponin, cancer	7-day mortality	0.804 (95%CI: 0.763–0.840)	Split-sample +bootstrap+ geographic validation (98, 99)	HL test *p*-value: 0.935	Web-based calculator
Daily HF score (34), 2013	921	1,310	OFISSER study (OS); Italian Clinical Service Project (OS); CONNECT study (IS); PARTNERS-HF study (OS); FAST study (IS); PRECEDE-HF study (OS); SENSE-HF study (OS)	America; Europe, Australia	Bayesian Belief Network	Intra-thoracic impedance, AF, RR during AF, %CRT pacing, ventricular tachycardia, night HR, HR variability,	Occurrence of HFH in the next 30 days	⋆	Geographic validation	NA	Risk score
HFPSI (35), 2013	1,536	445 486	Outpatient clinics (OS)	Michigan;	Cox proportional hazards analysis	BUN, BNP, NYHA class, diabetes, AF/atrial flutter, all-cause hospitalization within the prior 1 and 2–6 months	6-month risk of death and/or all-cause medical hospitalization	0.71	Geographic validation	NA	Risk score
GWTG-HF (Eapen) (36) , 2013	33,349	NA	GWTG-HF study (IS)	America;	Logistic regression	BUN, SBP, age, RR, BNP, HR, troponin, Cr, sodium, weight, hemoglobin, race; hemoglobin, sodium, SBP, HR, BUN, age, troponin, BNP, Cr, race; SBP, BUN, sodium, hemoglobin, HR, sodium, troponin, BNP, potassium, weight, RR, Cr;	30-day mortality after admission; 30-day rehospitalization after discharge; 30-day mortality/ rehospitalization after discharge	0.75 0.59 0.62	Geographic validation (97)	Calibration plot	Risk score
MAGGIC (37), 2013	39,372	NA	31 cohort studies (six randomized clinical trials and 24 observational registries)	Almost all major geographic regions;	Poisson regression	Age, LVEF, NYHA class, Cr, diabetes, β-blocker, SBP, BMI, HF duration, current smoker, COPD, sex, ACEI/ARB	1/3-year probability of death	⋆	Subgroup validation+ geographic validation (59, 100)	Calibration plot	Web-based calculator
3C-HF (38) , 2013	2016	4,258	Patients recruited at discharge or in the outpatient clinic (OS)	Europe	Logistic regression	Age, NYHA class, LVEF, renin–angiotensin system inhibitors, severe valvular heart disease, AF, β-blocker, chronic renal insufficiency, diabetes mellitus with target organ damage, anemia	1-year all-cause mortality	0.82 (95%CI: 0.81–0.83)	Bootstrap+ geographic validation	Calibration plot	Risk score
Redin-SCORE (39), 2015	2,507	992	Ambulatory patients (OS); MUSIC study	Spain	Fine-Gray regression	BNP, LV HF signs, eGFR; BNP, anemia, left atrial size, HR, LV HF signs, eGFR;	1-month and 1-year risk of readmission for worsening of HF in ambulatory patients	0.72/0.66	Bootstrap+ temporal validation	Calibration plot	Risk score
AHEAD (40) , 2016	5,846	6,315	AHEAD study (OS); GREAT study (OS);	Czech Republic, Italy, Spain, France, Argentina, Finland, Switzerland, USA, Tunisia, Austria	Logistic regression	AF, hemoglobin, age, Cr, diabetes	1-year all-cause mortality	0.639	Temporal validation	NA	Risk score
MEESSI-AHF (41) , 2017	4,867	3,229	Patients presenting to ED with ADHF	Spain	Logistic regression	Barthel index score at admission, SBP, NT-proBNP, age, potassium, troponin, NYHA class, RR, low-output symptoms, oxygen saturation, episode associated with ACS, hypertrophy on ECG, Cr	30-day mortality	0.828 (95%CI: 0.802- 0.853)	Temporal validation	HL test *p*-value: 0.122	Web-based calculator
AHFRS (42) , 2017	104	141	Hospitalized patients (OS);	Athens; Greece	Logistic regression	Hypertension, MI, red cell distribution width	1-year all-cause mortality or HF-rehospitalization	0.82 (95%CI: 0.73–0.89)	Temporal validation	NA	Risk score
Singapore HF risk score (43), 2019	1,392	729 804	SCDB-HF registry (IS)	Singapore;	Cox proportional hazards analysis	Age, MI, stroke, AF, peripheral vascular disease, SBP, QRS duration, LVEF, Cr, sodium	2-year all-cause mortality	0.68 (95% CI: 0.64–0.72),	Temporal validation	HL test *p*-value: 0.073	Web-based calculator
ESSIC-FEHF (44), 2020	762	916	ESSIC study (OS); AHFRS study (OS)	Spanish; Basque	Cox proportional hazards analysis	Age, SBP, sodium, LVEF, BUN, right ventricular failure	2-mouth all-cause mortality after the first episode of AHF	0.800 (95% CI: 0.724–0.876)	Temporal validation+ geographic validation	Greenwood-Nam-D'Agostino method	Risk score
MARKER-HF (45), 2020	1,986	1,956 888	Institutional electronic medical record (OS); BIOSTAT-CHF study (IS)	California;	ML	DBP, Cr, BUN, hemoglobin, white blood cell count, platelets, albumin, red blood cell distribution width	All-cause mortality	0.88 (95%CI: 0.85–0.90)	Geographic validation (101)	NA	Risk score (BDT algorithm)
MODEL 3 (46), 2025	2,257	348 388	Hospitalized patients (OS)	Paris; Lille	LASSO regression	ACEI, age, alkaline phosphatase, antibacterial agent, ARB, aspartate aminotransferase, bicarbonate, bilirubin, β-blocker, blood transfusion, BNP, cerebrovascular disease, creatine kinase, C-reactive protein, difference between SBP and DBP, diuretics, dyspnea, gamma-glutamyl transferase, hemoglobin concentration, heparin, hs-TnT, liver disease, low-density lipoprotein cholesterol, mean corpuscular hemoglobin concentration, mean corpuscular volume, obesity, oxygen saturation, pacemaker, platelet count, potassium, QSR duration, renal disease, SBP, sulfonamides, thyroid-stimulating hormone, transfer from another hospital, trimester of hospitalization, urea, urinary potassium, weight, severe dyspnea	90 day mortality in elderly patients with AHF	0.817 (95% CI: 0.789–0.845)	Temporal validation+ geographic validation	Calibration plot	Implemented in the hospital information system

NA, not applicable; IS, interventional study; OS, observational study; HL, Hosmer–Lemeshow; CBC, community-based cohort; EFFECT, Enhanced Feedback for Effective Cardiac Treatment; RR, respiratory rate; SBP, systolic blood pressure; BUN, blood urea nitrogen; COPD, chronic obstructive pulmonary disease; CART, classification and regression tree; CHARM, Candesartan in Heart Failure, assessment of reduction in mortality and morbidity; LVEF, left ventricular ejection fraction; BMI, body mass index; HFH, heart failure hospitalization; DBP, diastolic blood pressure; CHF, congestive heart failure; MI, myocardial infarction; HR, heart rate; AF, atrial fibrillation; CV, cardiovascular; HF, heart failure; OPTIMIZE-HF, Organized Program to Initiate Lifesaving Treatment in Hospitalized Patients with Heart Failure; OPTIME-CHF, Outcomes of a Prospective Trial of Intravenous Milrinonefor Exacerbations of Chronic Heart Failure; ADHERE, Acute Decompensated Heart Failure National Registry; Cr, creatinine; GWTG-HF, American Heart Association Get With the Guidelines-Heart Failure; PROTECT, Pulmonary Artery Catheterization and Hemodynamic Therapy in Heart Failure; VERITAS, Value of Endothelin Receptor Inhibition With Tezosentan in Acute Heart Failure Studies; ED, emergency department; ADHF, acute decompensated heart failure; EMS, emergency medical services; OFISSR, OptiVol Fluid-Index InSync Sentry Registry; CONNECT, Clinical Evaluation of Remote Notification to Reduce Time to Clinical Decision; PARTNERS HF, Program to Access and Review Trending Information and Evaluate Correlation to Symptoms in Patients With Heart Failure; FAST, Fluid Accumulation Status Trial; SENSE-HF, Sensitivity of the InSync Sentry OptiVol feature for the prediction of Heart Failure; CRT, cardiac resynchronization therapy; HFPSI, Heart Failure Patient Severity Index; BNP, brain natriuretic peptide; ACEI, angiotensin-converting enzyme inhibitor; ARB, angiotensin II receptor blocker; MUSIC, MUerte Súbita en Insuficiencia Cardíaca; LV, left ventricle; AHEAD, Acute Heart Failure Database; GREAT, Global Registry on Emergency Acute Heart Failure; NT-proBNP, N-terminal pro-brain natriuretic peptide; ACS, acute coronary syndrome; SCDB-HF, Singapore Cardiac Databank Heart Failure registry; ESSIC, Development of Clinical Prediction Rules and Health Services Research in patients with Heart Failure study; AHFRS, Validity of Scales to Assess Severity in Acute Heart Failure cohort; BIOSTAT-CHF, Biology Study to Tailored Treatment in Chronic Heart Failure; ML, machine learning; BDT, boosted decision tree; LASSO, least absolute shrinkage and selection operator. ⋆, While the C-statistic was not directly reported, the model's capacity for risk discrimination had been established by alternative approaches, including Kaplan–Meier curves.

Regarding modeling approaches, Cox proportional hazards regression was the most frequently employed (52%), followed by logistic regression (28%). A minority of studies used methods such as Fine-Gray competing risks regression, Bayesian belief networks, classification and regression trees, least absolute shrinkage and selection operator (LASSO) regression, and machine learning (ML). The predictors incorporated in the models spanned multiple categories including demographic information, signs/symptoms, comorbidities, medical history related to HF, laboratory data, electrocardiogram data, echocardiographic data, devices, and medications, ranging from a minimum of 3 to a maximum of 41. In terms of predicted outcomes, 30 (65%) models predicted all-cause mortality, 5 (11%) predicted HF rehospitalization, 1 (2%) predicted cardiovascular death, and 10 (22%) predicted composite outcomes.

Model discrimination was predominantly assessed using the concordance statistic (C-statistic), with values ranging from 0.62 to 0.88. Only two prediction models evaluated their discrimination ability using other methods, such as Kaplan–Meier curves. Regarding prediction model calibration, 37 (80%) prediction models underwent calibration assessment. Among these, 24 (65%) were evaluated through calibration plots, 12 (32%) were assessed through Hosmer–Lemeshow test *p*-values, and 1 (3%) was evaluated through the Greenwood–Nam–D'Agostino method. Only three models further provided DIC or NRI analysis (20, 24, 26). All models underwent external validation, either temporal or geographical, while 20 (20/46) also underwent internal validation. In terms of model presentation, 14 (30%) prediction models were presented as web-based calculators, 26 (57%) as risk scores, 5 (11%) as nomograms, and 1 (2%) prediction model was implemented in the hospital information system for application.

### Risk of bias assessment of included prediction models

The risk of bias for 46 prediction models from 38 included studies was assessed using the PROBAST tool. Results showed that 10 (22%) models from 10 studies had a high risk of bias, 21 (45%) from 18 studies had an unclear risk of bias, and 15 (33%) from 10 studies had a low risk of bias (Table 6). Regarding study populations, bias primarily stemmed from certain cohorts excluding high-risk patients through exclusion criteria, thereby limiting model representativeness and hindering generalizability to broader populations. Furthermore, among prediction models for HFpEF, inconsistencies in diagnostic criteria across studies led to heterogeneous populations, further compromising model applicability. Within the predictor domain, all studies explicitly reported predictor definitions and assessment time points, resulting in generally low bias risk. Regarding the outcome domain, all studies clearly described outcome definitions, measurement methods, and evaluation intervals, yielding similarly low bias risk in this domain. The analysis domain presented the highest concentration of bias risks. Potential sources of bias included: failure to meet the requirement of at least 20 events per variable; inappropriate handling of missing data; reliance solely on univariate analysis for predictor selection; and lack of consideration for model overfitting.

**Table 6:** Risk of bias assessment based on PROBAST tool.

**Prediction model, year**	**Participant**	**Predictor**	**Outcome**	**Analysis**	**Overall**
**HFrEF**					
HFSS (9) , 1997	+	+	+	?	?
OPTIME-CHF (10) , 2004	+	+	+	?	?
SHFM (11) , 2006	+	+	+	?	?
ESCAPE (12) , 2010	+	+	+	?	?
SHOCKED (13) , 2012	+	+	+	–	–
BCN-Bio-HF (14), 2014	+	+	+	?	?
BIOSTAT-CHF risk score (15) , 2017	+	+	+	+	+
PREDICT-HF (16) , 2020	+	+	+	+	+
PARADIGM-HFrEF (17) , 2021	+	+	+	?	?
**HFpEF and HFmrEF**					
ARIC-HFpEF (18), 2017	+	+	+	+	+
3A3B (19), 2019	+	+	+	?	?
MEDIA-ECHO score (20), 2021	–	+	+	?	–
I-PRESERV (21), 2021	+	+	+	?	?
EMPEROR-Preserved (22) , 2022	+	+	+	?	?
PREDICT-HFpEF (23), 2024	–	+	+	+	–
MODEL 1 (24) , 2025	+	+	+	+	+
MODEL 2 (25) , 2025	+	+	+	–	–
APSELNH (26) , 2024	+	+	+	+	+
**All HF regardless of types**					
EFFECT-HF (27), 2003	+	+	+	?	?
ADHERE (28) , 2005	+	+	+	–	–
CHARM (29), 2006	+	+	+	?	?
OPTIMIZE-HF (30) , 2008	+	+	+	?	?
GWTG-HF (Peterson) (31), 2010	+	+	+	+	+
PROTECT 7-day (32) , 2012	+	+	+	+	+
EHMRG (33) , 2012	+	+	+	?	?
Daily HF score (34), 2013	+	+	+	–	–
HFPSI (35), 2013	+	+	+	?	?
GWTG-HF (Eapen) (36) , 2013	+	+	+	+	+
MAGGIC (37), 2013	+	+	+	+	+
3C-HF (38) , 2013	+	+	+	?	?
Redin-SCORE (39), 2015	+	+	+	+	+
AHEAD (40) , 2016	+	+	+	?	?
MEESSI-AHF (41) , 2017	+	+	+	?	?
AHFRS (42) , 2017	+	+	+	–	–
Singapore HF risk score (43), 2019	+	+	+	?	?
ESSIC-FEHF (44), 2020	+	+	+	–	–
MARKER-HF (45), 2020	+	+	+	–	–
MODEL 3 (46), 2025	+	+	+	–	–

PROBAST: Prediction Model Risk of Bias Assessment Tool; + indicates low risk of bias; - indicates high risk of bias; ? indicates unclear risk of bias.

#### Prediction models for HFrEF

As shown in Table 3, multiple prediction models were available for assessing all-cause mortality in patients with HFrEF, including the Outcomes of a Prospective Trial of Intravenous Milrinone for Exacerbations of Chronic HF (OPTIME-CHF) prediction model (10), the Seattle HF Model (SHFM) (11), the Evaluation Study of Congestive HF and Pulmonary Artery Catheterization Effectiveness (ESCAPE) prediction model (12), the Barcelona Bio-HF (BCN-Bio-HF) prediction model (14), Biology Study to Tailored Treatment in Chronic HF (BIOSTAT-CHF) risk score (15), and the PREDICT-HF prediction model (16).

The OPTIME-CHF prediction model (10) was employed to predict short-term (60-day) mortality. However, this model was primarily developed in American patients with systolic dysfunction, limiting its applicability to those with diastolic dysfunction and non-American populations. External validation in a Chinese patient cohort demonstrated its poor predictive performance (47). Similarly, the ESCAPE model (12) predicted 60-month all-cause mortality, but it was derived from an interventional cohort that excluded some high-risk patients, thereby limiting its broader applicability.

In terms of long-term mortality prediction (1-year, 2-year, and 3-year), the SHFM, BCN-Bio-HF, BIOSTAT-CHF risk score, and PREDICT-HF all demonstrated predictive capability. Both the SHFM (11) and the PREDICT-HF (16) prediction models were developed using multi-regional global cohorts, affording them relatively wide applicability. The SHFM, in particular, was one of the most widely used models in clinical practice, partly due to the availability of a web-based calculator. Nevertheless, as an earlier model, the SHFM did not include key predictors such as B-type natriuretic peptide (BNP), and the absence of certain demographic variables may have compromised its predictive accuracy (48). Studies have also indicated that the SHFM may underestimate mortality in Black people individuals, those aged ≥65 years, and patients with implantable cardioverter defibrillators (ICDs) (49, 50). In contrast, the PREDICT-HF prediction model was developed in 2020 based on patients receiving guideline-directed medical therapy. It incorporated a comprehensive set of predictors spanning demographics, comorbidities, laboratory parameters, and medication use, contributing to its robust predictive performance. Notably, it included both BNP and angiotensin receptor–neprilysin inhibitor (ARNI) as predictive variables, making it particularly suitable for HFrEF patients receiving contemporary standard treatment. However, since the derivation cohort excluded ICD recipients, the model's applicability in such patients remained uncertain. Additionally, the model provided only a risk score without an online calculation tool, which limited its clinical convenience. Using the PREDICT-HF prediction model, the BIOSTAT-CHF risk score (15) was specifically designed for patients not receiving standard therapy, predicting all-cause mortality using just five simple variables. However, as this model was developed in European patients, further validation in broader regions remained necessary. Both the PREDICT-HF prediction model and BIOSTAT-CHF prediction model could also predict HF hospitalization (HFH) and composite endpoints. The BCN-Bio-HF prediction model (14) enhanced predictive performance by incorporating three emerging biomarkers—BNP, high-sensitivity cardiac troponin T (hs-cTnT), and soluble toll-like receptor-2 (ST2)—in addition to conventional predictors. This model was also derived from a guideline-treated cohort and was supported by an online calculator, facilitating its clinical dissemination. In recent years, researchers have integrated updated biomarker and treatment data to develop a refined version, BCN-Bio-HF prediction model 2.0, which has been widely applied (51).

A head-to-head comparative study (52) of contemporary HF prediction models evaluated the performance of BCN-Bio-HF 2.0, SHFM, and PREDICT-HF in predicting mortality among 1,166 HF outpatients. Results indicated that no single model demonstrated superior performance across all metrics. Among them, BCN-Bio-HF showed the best discriminative ability and overall performance, albeit with a tendency to overestimate mortality risk. In contrast, both SHFM and PREDICT-HF were observed to underestimate mortality risk.

The HF survival score (HFSS) (9) was employed to assess whether patients with end-stage HF require cardiac transplantation. This model categorized patients into three risk tiers through risk scoring: moderate-to-high risk patients, with a higher probability of death within 1 year, were recommended for cardiac transplantation within that timeframe, whereas low-risk patients could defer transplantation. However, BNP, a crucial prognostic predictor for HF, was not incorporated into the model. Subsequent studies reported that the addition of BNP to the HFSS enhanced the predictive capacity of the model (53, 54). With advances in medical care, guideline-recommended therapies including β-blockers, ICD, and cardiac resynchronization therapy (CRT) were widely applied after the creation of the model; although there were small sample sizes of studies showing that HFSS performed well in those populations, further validation was still required (55, 56).

The Prospective Comparison of ARNI With ACEI to Determine Impact on Global Mortality and Morbidity in HF (PARADIGM-HFrEF) prediction model (17) was employed to assess patients' risk of sudden cardiac death or pump failure, thereby guiding decisions regarding ICD implantation. Developed using a cohort spanning multiple countries worldwide, this model demonstrated considerable universality. However, as its modeling data primarily originated from interventional studies subject to stringent exclusion criteria, certain high-risk patients remained excluded. Consequently, the model exhibited limitations in its applicability. The SHOCKED prediction model (13) was specifically designed to predict 4-year all-cause mortality in HF patients with implanted ICDs, addressing the shortcomings of most previous models that did not cover this population. Unlike the PARADIGM-HFrEF model, the SHOCKED model was constructed from observational study cohorts with fewer excluded cases, thereby offering greater clinical applicability. However, this model still required further validation in populations outside the United States.

#### Prediction models for HFmrEF

##### APSELNH prediction model

As shown in Table 4, the APSELNH prediction model (26) predicted the 1-, 2-, and 3-year all-cause mortality risk in HFmrEF patients. The model achieved C-statistics above 0.8 at all time points, indicating good predictive performance. The model was ultimately presented as a nomogram, and the risk score could be calculated on the online platform. Patients could be categorized into three classes based on the risk score: low risk (point < 219.5), moderate risk (219.5 ≤ point ≤ 304.2), and high risk (point >304.2). Individualized risk stratification could guide treatment intensity and follow-up frequency, thereby enhancing the management of high-risk patients while appropriately reducing unnecessary interventions in low-risk patients. However, as the model was derived exclusively from Chinese patients, external validation in more diverse regions and ethnic groups was warranted. Furthermore, key biomarkers such as troponin I/T (TnI/T) were not included during model development, representing a potential direction for future refinement.

#### Prediction models for HFpEF

As shown in Table 4, Atherosclerosis Risk in Communities (ARIC-HFpEF) (18), MODEL 1 (24), and the 3A3B risk score (19) were three prediction models that could be used to predict all-cause mortality in patients with HFpEF. Among them, the ARIC-HFpEF prediction model (18) was capable of predicting both short- (28-day) and long-term (1-year) mortality risk. Notably, it was the first prediction model specifically developed for patients with acute decompensated HFpEF (LVEF ≥50%), who typically presented with poorer prognoses. During its development, the model innovatively incorporated variables related to signs/symptoms, such as shortness of breath, edema, and hypoxia, with hypoxia ultimately being established as a formal predictor. Additionally, the model was presented as a web-based calculator, greatly facilitating its clinical application. MODEL 1 (24) was used to predict 3-year mortality in HFpEF patients (LVEF ≥50%) and included a series of novel predictors related to inflammation and nutritional status, though it did not incorporate treatment-related variables. The 3A3B risk score (19) aimed to predict 5-year mortality risk in HFpEF patients (LVEF ≥50%). Constructed based on an observational cohort with few exclusions, this model demonstrated good applicability. However, all three models were developed using cohorts from single regions, and their generalizability required further external validation in other populations. Similar to the PARADIGM-HFrEF prediction model, the Irbesartan in HF with Preserved Ejection Fraction Study (I-PRESERVE) prediction model was employed to predict sudden death and pump failure in patients with HFpEF (LVEF >40%, LVEF ≥45%). However, this model did not incorporate BNP as a predictor and also exhibited shortcomings in calibration.

On the other hand, the European MEtabolic Road to DIAstolic HF (MEDIA) echo score (20), Empagliflozin Outcome Trial in Patients with Chronic HF and a Preserved Ejection Fraction (EMPEROR-Preserved) (22), PREDICT-HFpEF (23), and MODEL 2 (25) were models used to predict either HFH or a composite outcome of HFH and cardiovascular death. Echocardiography played a crucial role in the diagnosis and prognosis assessment of HFpEF. The MEDIA echo score (20) could predict the composite outcomes in HFpEF patients (LVEF >50%, LVEF ≥45%) by incorporating echocardiographic parameters [pulmonary artery systolic pressure, inferior vena cava collapsibility, early diastolic mitral inflow velocity/early diastolic mitral annular tissue velocity (E/e′), and lateral s′] into traditional clinical predictors. Subsequent studies had confirmed that this model demonstrated good predictive performance in both outpatient and inpatient settings. The EMPEROR-Preserved (22) and PREDICT-HFpEF models (23) also assessed the risk of composite outcomes in HFpEF patients (LVEF >45%) via online web-based calculators. Since both models were developed using cohort data from multiple regions worldwide, they provided high applicability. However, as both models were based on interventional study cohorts that excluded some high-risk populations, their predictive performance in high-risk groups still required further validation. It was worth noting that the EMPEROR-Preserved model included key predictors such as N-terminal pro-brain natriuretic peptide (NT-proBNP) and hs-cTnT, whereas the newly developed PREDICT-HFpEF model was the first to incorporate sodium–glucose cotransporter 2 inhibitor (SGLT2i) as a predictive variable. The MODEL 2 (25) predicted the risk of HFH within 1 year in HFpEF patients (LVEF ≥50%) through a web-based dynamic nomogram. It included important variables such as BNP, LVEF, E/e′, and the use of angiotensin-converting enzyme inhibitor (ACEI)/angiotensin II receptor blocker (ARB)/ARNI, but did not include SGLT2i, indicating room for further refinement in the future.

It was worth noting that the LVEF thresholds for defining HFpEF in the selected data cohorts of various prediction models were not consistent, ranging from 40 to 45 to 50. This may be due to the fact that some of the studies were earlier, and at that time, there was not yet a uniform and clear delineation of HFpEF. Studies have shown significant differences in baseline characteristics, incidence, and prognosis between HFpEF and HFrEF populations (48). It is suggested that future predictive modeling studies should pinpoint LVEF at 50% when selecting study populations. In addition, previous prediction models (I-PRESERVE, the MEDIA echo score, EMPEROR-Preserved, PREDICT-HFpEF) need to be validated in populations with LVEF ≥50% to further assess their predictive efficacy. Furthermore, as HFpEF patients have been further subdivided into stages A/B/C/D, more researchers have focused on developing predictive models to prevent patients' progression from stage A/B (where HF symptoms have not developed) to stage C/D (where HF has occurred), advancing the front line of prevention and treatment to improve patients' prognosis (57, 58).

#### Prediction models for all HF regardless of types

As shown in Table 5, a total of 22 prediction models are currently available for assessing the prognosis of all HF patients regardless of subtype, with prediction timeframes ranging from the inpatient period up to 3 years. The American Heart Association Get With the Guidelines-HF (GWTG-HF) (Peterson) model (31) is suitable for predicting in-hospital mortality. GWTG-HF (Eapen) (36), Enhanced Feedback for Effective Cardiac Treatment (EFFECT-HF) (27), and Daily HF score (34) were employed to forecast 30-day mortality, readmission, and their composite endpoints. Specifically, GWTG-HF (Eapen) (36) is more applicable to HF patients over 65 years of age, EFFECT-HF (27) is more suitable for hospitalized HF patients, and the Daily HF score (34) is better suited for prognostic assessment in outpatients with implanted pacemakers. None of these models incorporated BNP, and they still require further external validation in broader geographical regions. Additionally, as the Daily HF score was derived from pacemaker data, it did not include demographic or medication-related variables.

The Organized Program to Initiate Lifesaving Treatment in Hospitalized Patients with HF (OPTIMIZE-HF) (30), Redin-SCORE (39), the Development of Clinical Prediction Rules and Health Services Research in Patients with HF study (ESSIC-FEHF) (44), MODEL 3 (46) and HF patient severity index (HFPSI) (35) prediction models were employed to forecast all-cause mortality or HFH over periods ranging from 1 to 6 months. The OPTIMIZE-HF prediction model (30) was developed to predict mortality within 60–90 days post-discharge, while the ESSIC-FEHF prediction model (44) was employed to forecast mortality within 2 months; however, neither of these models incorporated BNP. Both Redin-SCORE and HFPSI prediction models were developed using outpatient cohorts: Redin-SCORE (39) predicted 1-month mortality, while the HFPSI prediction model (35) forecasts 6-month all-cause mortality or HFH. MODEL 3 (46) was a predictive model developed using LASSO regression on EHR data to forecast 90-day mortality in elderly HF patients. This model could be directly implemented in the hospital information system for intelligent prediction of HF patients. All three models incorporated BNP but required further external validation in other countries and regions. Additionally, the EFFECT-HF and Redin-SCORE prediction models could predict 1-year all-cause mortality.

For long-term prognosis prediction, the Meta-Analysis Global Group In Chronic HF (MAGGIC) (37), 3C-HF (38), Singapore HF risk score (43), MARKER-HF (45), and Candesartan in HF: A Randomized Multinational Strategy Trial (CHARM) (29) prediction models were employed to forecast all-cause mortality over 1–3 years. The MAGGIC prediction model (37) remained the most widely employed model for predicting 1- or 3-year mortality. Its strengths lie in its construction from cohorts across multiple global regions and the provision of an online web calculator, demonstrating outstanding clinical applicability and convenience. Consequently, it has become the most extensively utilized model in clinical practice and research. However, this model excluded BNP, potentially leading to an overestimation of mortality risk (52). Research indicated that MAGGIC demonstrated poor predictive performance in Japanese cohorts; however, incorporating BNP into the model enhanced its predictive performance (59). The Singapore HF risk score (43) was a 2-year mortality prediction model specifically developed for East Asian HF patients; the CHARM prediction model (29) could predict a composite endpoint of cardiovascular death, all-cause mortality, and HFH within 2 years; the MARKER-HF prediction model (45) was an all-cause mortality prediction model built using ML methods, suitable for individuals under 80 years of age.

In addition to the prediction models mentioned above, several prediction models were specifically designed for patients with acute decompensated HF (ADHF). The Emergency HF Mortality Risk Grade (EHMRG) (33) and the Multiple Estimation of Risk based on the Emergency Department Spanish Score In Patients with AHF (MESSI-AHF) risk score (41) were models that could be rapidly applied in the emergency department (ED) to assist in determining whether an ADHF patient required hospitalization. The EHMRG prediction model (33) primarily predicted 7-day mortality, while the MESSI-AHF risk score (41) predicted 30-day mortality. In terms of predictor selection, both models utilized clinical variables readily available in the ED. The MESSI-AHF risk score incorporated NT-proBNP and troponin as predictors. Validation results demonstrated that even without NT-proBNP, these two models could maintain retain a certain level of predictive performance; however, the inclusion of NT-proBNP further enhanced their predictive accuracy. Considering the comprehensiveness of predictors, the rigor of validation methodologies, and the representativeness of study populations, MESSI-AHF demonstrated superior predictive performance and greater potential for clinical applicability.

The Acute HF Risk Score (AHFRS) (42) was also a prediction model established for patients presenting to the ED. In contrast to the two prediction models mentioned above, it was designed to predict the risk of 1-year all-cause mortality or HFH for patients who received guideline-directed medical therapy during hospitalization and after discharge. This model provided a long-term prognostic assessment for ADHF patients admitted to the ED.

Furthermore, several prediction models were available to assess the prognosis of ADHF patients who had already been hospitalized. The Acute Decompensated HF National Registry (ADHERE) risk tree (28) was constructed to predict in-hospital mortality. By judging blood urea nitrogen (BUN) levels (threshold: 43 mg/dl), systolic blood pressure (SBP; threshold: 115 mm Hg), and serum creatinine levels (threshold: 2.75 mg/dl) sequentially, ADHF patients were readily categorized into low-, intermediate-, and high-risk groups for in-hospital death, with the risk of death ranging from 2.1 to 21.9%. The Pulmonary Artery Catheterization and Hemodynamic Therapy in HF (PROTECT) 7-day model (32) predicted a 7-day composite outcome of death, HFH, or worsening HF in ADHF patients with renal dysfunction and elevated BNP levels. The AHEAD prediction model (40) also constructed risk scores to predict 1-year all-cause mortality. However, BNP was not included in any of the above models, probably because these studies were limited by the research context at the time and the records related to serum markers were incomplete. However, serum markers played an increasingly important role in the treatment and diagnosis of HF. A predictive model based on ML had screened a biomarker cluster to predict the prognosis of HF patients, and its predictive performance was even better than MAGGIC prediction model (60). This phenomenon was worthy of consideration.

## Discussion

Due to the heterogeneity of HF, researchers have developed different prediction models for different types of HF. Prediction models for HFrEF and all HF types are more mature, while the number of studies on HFpEF and HFmrEF are fewer due to the newer concepts and definitions, although related studies are gradually growing (61). This study comprehensively summarizes the characteristics and shortcomings of each prediction model and visually presents them in the form of tables (Tables 7–9). Among them, the prediction models in bold are considered to have better predictive performance than the others.

**Table 7:** Advantages and disadvantage of prediction models for HFrEF.

**Prediction model, year**	**Advantages**	**Disadvantages**
HFSS (9) , 1997	Recommendations for further treatment can be made based on the level of risk score	The role of β-blockers in prediction was not considered
OPTIME-CHF (10), 2004	The baseline data of the patients in this study cohort were relatively comprehensive	Poor predictive performance in patients with chronic HF with predominantly diastolic function and in non-US patients
SHFM (11), 2006	Had good cross-regional and cross-population applicability; The model could estimate the impact of adding drugs or devices to a patient's treatment regimen on survival; An online calculator had been developed	Underestimated the risk of events at some point; The SHFM was developed and validated in outpatients; Patients with serious life-altering comorbidities such as cirrhosis, renal failure, dementia or cancer were excluded
ESCAPE (12), 2010	The model could distinguish severe HFrEF patients and thus provided more intensive monitoring and more advanced treatments	Patients with a clearly poor prognosis were excluded in the research The model validated in the external queue missing two indicators
SHOCKED (13), 2012	The model was built on observational studies where the population was not selective, making this model closer to real-world situations	This study did not further explore the benefit of ICD therapy in low-risk patients
BCN-Bio-HF (14), 2014	The majority of the population included in this study received evidence-based pharmacological treatment; The model was updated to incorporate novel indicators, including ARNI, resulting in enhanced generalizability	Further validation was needed for inpatients and large cohorts from different regions and ethnicities; ST2 may be uncommon in clinical tests
BIOSTAT-CHF risk score (15), 2017	This model aimed to predict the need for future ICD implantation; The model was derived from a multi-racial and multi-regional patient cohort under evidence-based care, which exhibited robust generalization capability	Some specific patients were excluded in RCT cohorts, which would affect the application of the model to these patients
PREDICT-HF (16), 2020	An online calculator had been developed; Provide prognosis prediction for patients who had not received standardized treatment	The study population was predominantly European, necessitating further validation in other regions
PARADIGM-HFrEF (17), 2021	Accurately reflect the impact of race and geographic factors on prognosis; The included population received evidence-based treatments	The datasets used in this model excluded some populations, such as those with reduced renal function; ICD, CRT, functional capacity and frailty were not assessed in the model

HF, heart failure; US, United States; HFrEF, heart failure with reduced ejection fraction; ICD, implantable cardioverter-defibrillator; ARNI, angiotensin receptor–neprilysin inhibitor; ST2, soluble toll-like receptor-2; RCT, randomized controlled trial; CRT, cardiac resynchronization therapy.

**Table 8:** Advantages and disadvantage of prediction models for HFpEF and HFmrEF.

**Prediction model, year**	**Advantages**	**Disadvantages**
ARIC-HFpEF (18), 2017	Focusing on the prognosis of acute decompensated HFpEF patients; Added HF signs and symptoms (such as shortness of breath, edema and hypoxia) to the variable screening; A web-based calculator is available	This predictive score was only for hospitalizations
3A3B (19), 2019	It was the first risk score to predict long-term prognosis (5-year all-cause mortality); The selected observational studies included HFpEF patients without any exclusion criterion other than < 20 years old	Other important prognostic factors were not considered, such as COPD
MEDIA-ECHO score (20), 2021	BNP and echocardiographic parameters were included in the score	There was no web-based calculator that is convenient for computation; The validation cohort (LVEF ≥45%) was somewhat inconsistent with the currently defined HFpEF population, which necessitates further external validation
I-PRESERV (21), 2021	This model aimed to predict the need for future ICD implantation	The model was primarily derived from a White population and required validation in other racial and ethnic groups
EMPEROR-Preserved (22), 2022	A web-based calculator is available; The model simultaneously incorporated two important biomarkers: hs-cTnT and NT-proBNP	This model is not applicable to patients who had ADHF in the past week or a recent MI in the past 3 months
PREDICT-HFpEF (23), 2024	Based on large samples, wide geographic and race ranges of RCT cohorts; An online calculator has been developed	Some specific patients were excluded in RCT cohorts, which would affect the application of the model to these patients
MODEL 1 (24), 2025	In addition to conventional clinical parameters, nutritional and inflammatory indicators were incorporated to allow for a comprehensive assessment of patient status	Treatment factors were not included as candidate predictors
MODEL 2 (25), 2025	Web-based dynamic nomogram was provided	SGLT2i were not included as candidate predictors
APSELNH (26), 2024	This study recruited only HFmrEF patients; Online calculator was provided	This study only conducted in Chinese patients; Troponin I/T, arterial blood gas analysis and CHA2DS2-VASc score were not considered

HFpEF, heart failure with preserved ejection fraction; HF, heart failure; COPD, chronic obstructive pulmonary disease; BNP, brain natriuretic peptide; ICD, implantable cardioverter-defibrillator; hs-cTnT, high-sensitivity cardiac troponin T; NT-proBNP, N-terminal pro-brain natriuretic peptide; ADHF, acute decompensated heart failure; MI, myocardial infarction; RCT, randomized controlled trial; SGLT2i, sodium–glucose cotransporter 2 inhibitors; HFmrEF, heart failure with mildly reduced ejection fraction.

**Table 9:** Advantages and disadvantage of prediction models for all HF regardless of types.

**Prediction model, year**	**Advantages**	**Disadvantages**
EFFECT-HF (27), 2003	The variables included in the model were easily obtained at the early hours of admission; The model could predict both short- and long-term prognosis of hospitalized HF patients; The model was built on observational studies	This study was conducted on hospitalized patients and may not be directly applicable to outpatient HF patients
ADHERE (28), 2005	Variables were clinically accessible	Medication-related data were not included
CHARM (29), 2006	Variables were clinically accessible	Serum and treatment-related data were not considered; No external validation; The 21 predictors were a bit cumbersome, as well as were inconvenient in clinical application
OPTIMIZE-HF (30), 2008	Variables were clinically accessible	BNP was not included in the model
GWTG-HF (Peterson) (31), 2010	The model could broadly applicable to patients with various types of HF; Variables in the model were easy to collect in clinical practice	BNP was not included in the model
PROTECT 7-day (32), 2012	The prediction model included non-mortality-related outcomes, such as worsening HF, broadening the range of clinical applications of model	The model was limited in its generalizability to African Americans and women; BNP was not included in the model
EHMRG (33), 2012	To provide robust support for emergency physicians in evaluating decisions regarding patient admission or discharge; Variables were available in the ED; An online calculator had been developed	Patients who were palliative before ED arrival were excluded; BNP and LVEF were not included in the prediction model; xternal validation in healthcare systems beyond Canada was necessary to demonstrate its generalizability
Daily HF score (34), 2013	The model was derived from diagnostic parameters monitored in implantable devices, it could assessed the risk of HFH in outpatients with implanted devices	Most of the data included in the study was from within the first year of the device life, thus the results may not reflect the performance of the risk score during the later years of the device life
HFPSI (35), 2013	It was presented as a risk score, with defined risk categories for different score ranges	The model was developed in a relatively young HF patient population, further validation in broader and more diverse populations and regions was required
GWTG-HF (Eapen) (36), 2013	This model predicted the prognosis of elderly HF patients, in line with the growing number of elderly HF patients	The model's performance in predicting 30-day rehospitalization after discharge was relatively mediocre
MAGGIC (37), 2013	Enrolled the most comprehensive population, and the generalizability of the model to other populations was improved; An online calculator had been developed	BNP was not included as a model candidate variable; The model may overestimate mortality
3C-HF (38), 2013	The study cohort include HF patients with various degrees and complications; The LVEF cutoff for identifying HFpEF patients was 50%, rather than 40% as in most previous studies	Device therapy related data was not included; BNP was not routinely available in studies at that time
Redin-SCORE (39), 2015	This model provided prognostic prediction for HF patients in the outpatient setting	The model required further validation in other countries
AHEAD (40), 2016	Variables were clinically accessible	Cancer, COPD, hypotension, LVEF, hyponatremia, and hyperuricemia were not taken into account; Patients who were at high risk of mortality were excluded
MEESSI-AHF (41), 2017	The model incorporated biomarkers such as NT-proBNP and troponin, but it maintained robust predictive performance even when these values were missing; An online calculator had been developed	LVEF were not included in the prediction model; The model should be further validated in other countries and regions
AHFRS (42), 2017	Proposed a long-term (1 year) prediction model for ADHF	The number of patients in this study cohort was limited; AHF-related medication information was poorly documented
Singapore HF risk score (43), 2019	This risk score is the first risk score developed specifically for Asian HF patients; An online calculator had been developed	Due to about 15% of missing data, NT-proBNP was excluded from the model
ESSIC-FEHF (44), 2020	The model predicts the prognosis after a first episode of AHF	Other important prognostic factors were not considered, such as NT-proBNP, hs-TnT, and dyspnea
MARKER-HF (45), 2020	This model was developed using a machine learning approach and demonstrates superior predictive performance compared to the GWTG-HF (Peterson) or ADHERE risk scores	The model's development was based solely on HF patients under 80 years of age, which limits its applicability
MODEL 3 (46), 2025	This is the first model developed to predict 90-day post-discharge mortality for HF decompensation episodes using data from the EHR available up to 48 h after admission	The model required further validation in other countries

HF, heart failure; BNP, brain natriuretic peptide; ED, emergency department; LVEF, left ventricular ejection fraction; HFH, heart failure hospitalization; COPD, chronic obstructive pulmonary disease; NT-proBNP, N-terminal pro-brain natriuretic peptide; ADHF, acute decompensated heart failure; AHF, acute heart failure; hs-cTnT, high-sensitivity cardiac troponin T; EHR, electronic health record.

In earlier studies, HF types were relatively simple, mainly dominated by HFrEF, so the earliest prediction models focused mainly om HFrEF patients. With advances in research, it was found that the LVEF in some HF patients was not significantly reduced, which put forward the concept of diastolic HF (62, 63). However, the definition of LVEF for diastolic HF has long been controversial (>40%, >45%, or ≥50%) (20–23), posing significant challenges for prognostic prediction. In this context, general prediction models that do not distinguish HF types emerged, the most representative of which was the MAGGIC-HF prediction model. In recent years, with the clarification of HF classification criteria, diastolic HF has been formally defined as HFpEF by the guidelines (64, 65), and a specific LVEF cutoff (≥50%) has been established. Additionally, the guidelines introduced a new classification called HFmrEF. This refinement in classification has driven the development of prediction models for specific HF subtypes. Several studies (23, 66) have confirmed that prediction models based on specific HF subtypes are significantly better than MAGGIC-HF models (which do not distinguish types), suggesting the inherent limitations of unified prediction methods. At present, constructing prediction models for different HF subtypes has become the mainstream strategy to improve prediction accuracy and reduce the occurrence of adverse events.

**Figure 2:**
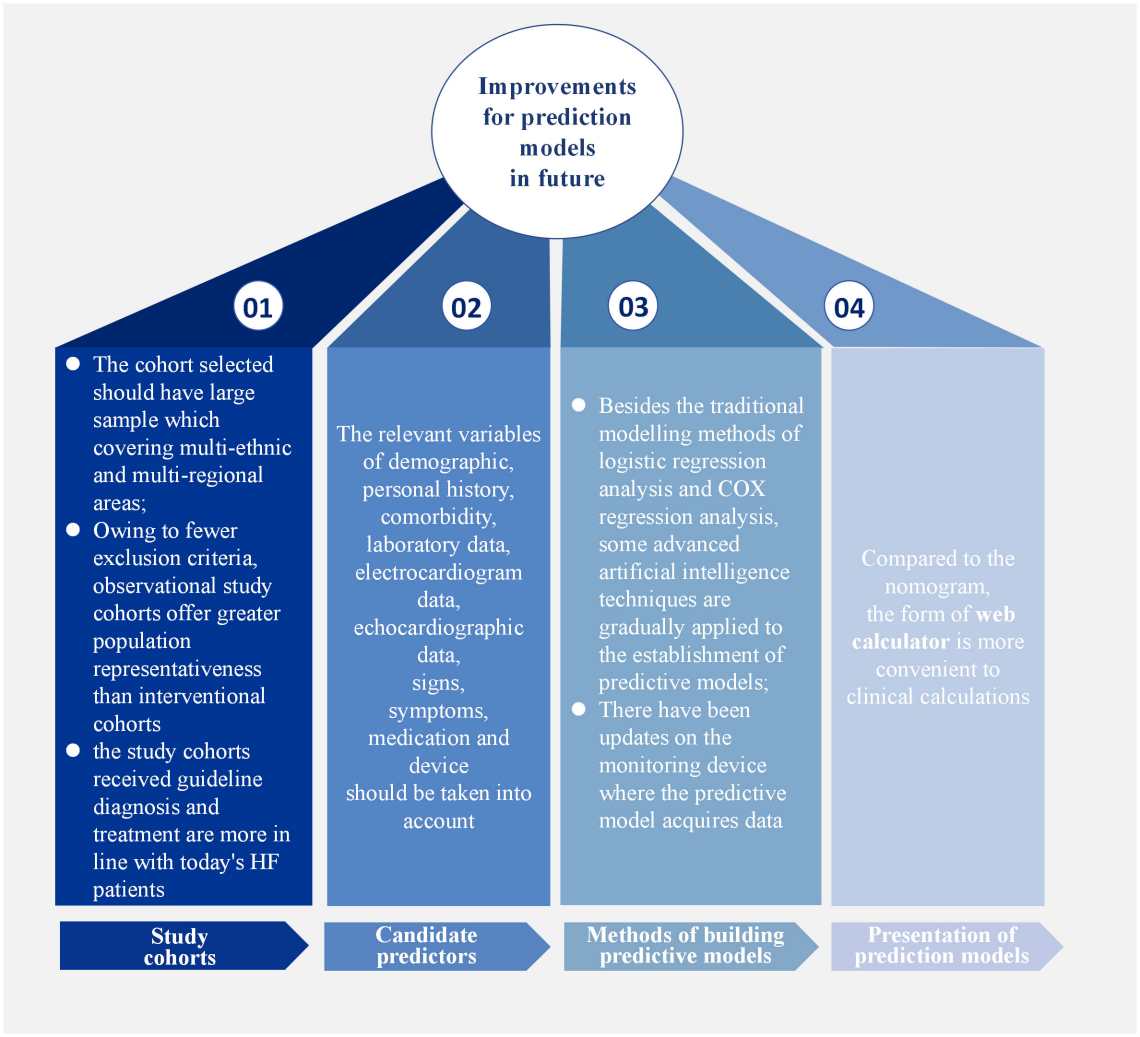
Improvements for prediction models in future.

Among the predictive models for different HF subtypes, those for HFrEF have been the most extensively studied. However, due to limitations in research conditions at that time, early models did not include certain predictors now regarded as essential, which restricted their applicability in contemporary clinical practice. For HFpEF prediction models, a major issue is that the LVEF criteria used in early studies do not align with current guidelines, making it difficult to directly apply these models to today's patient populations. In contrast, research on HFmrEF prediction models is relatively scarce; only one externally validated model was included in this review, underscoring the need for further exploration in this area. Notably, no single model currently dominates in terms of predictive efficacy or clinical applicability. This may be attributed to several factors, such as incomplete selection of study cohorts, insufficient candidate predictors, and inconvenient presentation of prediction models (Figure 2).

The study cohort was both source of research data and the basis for model construction, making the selection an appropriate study cohort crucial. Most of the study cohorts used in developing prediction models were small to medium-sized single-center cohorts. As these cohorts covered insufficient populations, representative predictors, such as race, region, and gender, could not be screened out. Prediction models established in this way also needed to be externally verified by multi-center studies and larger cohorts. In contrast, widely used prediction models, such as MAGGIC-HF and SHFM-HFrEF, selected large sample cohorts that included multi-ethnic and multi-regional areas to ensure their universal applicability to a broader patient population. Additionally, prediction models based on observational study cohorts are applicable to a wider range of populations than those based on intervention studies. Interventional studies inevitably excluded patients with a high mortality risk, such as those with hypoalbuminemia or cancer, or those with a recent history of myocardial infarction or HFH within the past 3 months. This not only limited the application of prediction models in these populations but also introduces bias into the models. While observational studies have fewer exclusion criteria and can include patients more comprehensively, the larger the number of intervention studies in the real world has led most of prediction models to rely on intervention studies as their modeling cohorts, thus limiting their applicability. Finally, study cohorts that have received guideline-recommended diagnostics and treatments are more suitable as target populations for developing prediction models. In recent years, significant progress has been made in the diagnosis and treatment of HF, and guideline recommendations for clinical practice have been established. Although earlier prediction models helped predict the prognosis of HF patients, biomarkers such as BNP/NT-proBNP and echocardiography-related indices were not regarded as important for HF diagnosis at that time, nor were β-blockers, ACEI, ARB, ARNI, and SGLT2i used as routine treatments. Subsequent studies have proven the predictive incremental effect of BNP/NT-proBNP on early prediction models (48, 59), indicating that the applicability of earlier prediction models in contemporary HF patients receiving guideline-based therapy remains limited. In contrast, the PREDICT-HFpEF and APSELNH-HFmrEF prediction models created in recent years, selected study populations that received standardized evidence-based treatment, and the final models included the novel diagnostic and therapeutic indices mentioned above, confirming the important role of these indices in HF prognosis.

Predictors play an essential role in the model, so their selection should be more comprehensive, taking into account relevant variables such as demographic information, personal history, comorbidities, laboratory data, electrocardiogram data, echocardiographic data, signs, symptoms, medication, and devices. All variables mentioned in the above prediction models are shown in Table 10. Prediction models such as I-PRESERVE, ADHERE, and 3C-HF illustrate that predictive performance can be limited by incomplete predictor selection. In recent years, numerous biomarkers for predicting HF prognosis have emerged, such as ST2, growth differentiation factor-15 (GDF-15), and microRNAs (67–69). Several studies have demonstrated that incorporating novel predictors into existing models enhances predictive performance (54, 70, 71). Furthermore, several studies (72, 73) have employed ML approaches to develop prognostic prediction models by integrating inflammatory biomarkers with clinical variables. These models have demonstrated good discriminatory performance, showing potential not only to assist in clinical decision-making but also to improve patient outcomes. Emerging detection technologies, including proteomics (74, 75), metabolomics (76, 77), genomics (78), and artificial intelligence-based imaging data analysis (79, 80) are progressively being applied to the construction of HF prognosis prediction models. It should be noted that most of these models have currently undergone only internal validation and have yet to be externally validated in independent cohorts. Their predictive efficacy and clinical applicability require further confirmation. Concurrently, the correlation between established clinical scoring tools—such as the CHA_2_DS_2_-VASc score (81) and H_2_FPEF score (82)—and HF prognosis has been substantiated, providing additional rationale for selecting potential predictive factors within forecasting models. Echocardiographic indicators have become increasingly important references in the diagnosis of HF (83, 84), especially HFpEF, but they are less frequently included in current prediction models, warranting more attention in the future. In addition, there have been updates on the monitoring devices from which predictive models acquire data. Recent studies have found a way to monitor the physiological information of HF patients remotely by minimally inserting a cardiac monitor under the skin, subsequently developing an HF risk score to predict the occurrence of worsening HF events, thus providing a multi-parameter and comprehensive prediction method (85, 86). However, the sample size of this study was small, and further verification is needed. The application of novel technologies has undoubtedly enhanced the real-time capability and accuracy of predictions. In developing future predictive models, the selection of predictive factors must take full account of the aforementioned considerations.

**Table 10:** Variables mentioned in the above prediction models.

**Predictors**	**Variables**	**Count**	**Ratio**	**Predictors**	**Variables**	**Count**	**Ratio**
Demographic	Age	29	63.04%	Laboratory data	BNP/NT-proBNP	25	54.35%
	Sex	8	17.39%		Blood urea nitrogen	16	34.78%
	Race	8	17.39%		Hemoglobin	11	23.91%
	Region	4	8.70%		Sodium	14	30.43%
Comorbidity	Diabetes	14	30.43%		Potassium	7	15.22%
	Atrial fibrillation	11	23.91%		Albumin	7	15.22%
	Ischemic cardiomyopathy	10	21.74%		LDL/HDL/ApoA-1/ApoB/total cholesterol/Triglyceride	6	13.04%
	Chronic obstructive pulmonary disease	8	17.39%		Estimated glomerular filtration rate	6	13.04%
	PTCA/PCI/CABG	5	10.87%		Troponin	5	10.87%
	Peripheral arterial disease	3	6.52%		Uric acid	4	8.70%
	Stroke	3	6.52%		Creatinine	3	6.52%
	Cancer	3	6.52%		Bilirubin	3	6.52%
	Valvular heart disease	2	4.35%		Bilirubin	3	6.52%
	Chronic renal insufficiency	2	4.35%		Lymphocytes	2	4.35%
	Bundle branch block	2	4.35%		Platelet count	2	4.35%
	Cerebrovascular disease	2	4.35%		Red cell distribution width	2	4.35%
	Dementia	1	2.17%		Neutrophils	2	4.35%
	Hepatic cirrhosis	1	2.17%		Creatine kinase isoenzyme	1	2.17%
	Hypertension	1	2.17%		Soluble toll-like receptor-2	1	2.17%
	Cardiomegaly	1	2.17%		High sensitive C-reactive protein	1	2.17%
	Mitral regurgitation	1	2.17%	Electrocardiogram data	Bundle branch block	4	8.70%
Medical history related to HF	Prior HF hospitalization	10	21.74%		QSR duration	3	6.52%
	HF duration	8	17.39%		Heart rate variability	1	
	Primary cause of admission	1	2.17%		Intraventricular conduction delay	1	2.17%
	Cardiopulmonary resuscitation or mechanical ventilation during hospitalization	1	2.17%	Echocardiographic data	Left ventricular ejection fraction	16	34.78%
Sign/symptom	SBP/DBP/BP	23	50.00%		E/e'	2	4.35%
	NYHA Class	17	36.96%		Inferior vena cava collapsibility	1	2.17%
	Heart rate	15	32.61%		Lateral s'	1	2.17%
	Body mass index	8	17.39%		Right atrial end-systolic diameter	1	2.17%
	Respiratory rate	5	10.87%		Left ventricular systolic dysfunction	1	2.17%
	Weight	4	8.70%		Left atrial size	1	2.17%
	Minnesota living quality of life (KCQQ)	3	6.52%	Others' exam data	Pulmonary capillary wedge pressure	1	2.17%
	Oedema	2	4.35%		Peak Vo2	1	2.17%
	6-minute walk	1	2.17%	Device	Cardiopulmonary resuscitation or mechanical ventilation during hospitalization	1	2.17%
	Pulmonary crackles	1	2.17%		Implanted cardiac defibrillator		
Medication	ACEI/ARB/ARNI	11	23.91%	Medication	Aldosterone blocker	1	2.17%
	β-blocker	11	23.91%		Blood pressure-lowering medication	1	2.17%
	Statin	2	4.35%		Sulfonamides	1	2.17%
	SGLT2i	1	2.17%		Allopurinol	1	2.17%

PTCA, percutaneous transluminal coronary angiograph; PCI, percutaneous coronary intervention; CABG, coronary artery bypass grafting; HF, heart failure; SBP, systolic blood pressure; DBP, diastolic blood pressure; BP, blood pressure; ACEI, angiotensin-converting enzyme inhibitors; ARB, angiotensin II receptor blocker; ARNI, angiotensin receptor–neprilysin inhibitor; SGLT2i, sodium–glucose cotransporter2inhibitors; BNP, brain natriuretic peptide; NT-proBNP, N-terminal pro-brain natriuretic peptide; LDL, low-density lipoprotein cholesterol; HDL, high-density lipoprotein cholesterol.

Moreover, the presentation of the model is equally important. Most models are presented in the form of computational formulas or nomograms, but the web calculator format is more conducive to clinical calculations, facilitating the wider dissemination of models. The bold models in the table are all in the form of web calculators. With advances in technology, predictive models using ML for EHR data have been gradually developed. These models could be implemented in the hospital information systems to intelligently identify the prognosis of in-hospital HF patients (46).

In addition to the traditional modeling methods of logistic regression analysis and COX regression analysis, some advanced artificial intelligence techniques have gradually been applied to the establishment of predictive models. For example, a growing number of researchers are leveraging ML algorithms (such as LASSO regression, light gradient-boosting machine, and extreme gradient boosting) for model development. The advantage of ML lies in its ability to train multiple candidate models in parallel and, through performance comparison, ultimately identify the model with the highest predictive accuracy (73, 87–89). The study by Jawadi et al. (90) reported that an ML-based model developed to predict in-hospital mortality in HF patients demonstrated superior discrimination, sensitivity, and specificity compared to traditional ADHERE and GWTG-HF prediction models (90). The study by Abdul-Samad et al. also indicated that the ML model exhibited better calibration capability than conventional statistical models (91). As discussed in the preceding section, ML's powerful computing capabilities provide a natural advantage for processing high-throughput, multi-modal data like omics and medical imaging. Given this capability, it is positioned as an ideal tool for developing such high-performance predictive models. However, most ML-based prediction models have only undergone internal validation and have not yet been translated into risk scoring systems ready for clinical application. This critical aspect requires improvement in future model development.

Last but not least, how clinical prediction models and risk scores can be better integrated with clinical treatment remains a worthy question for consideration. Although HF patients can be assessed as low, intermediate, or high risk through prediction models, clinical HF guidelines have not provided specific therapeutic recommendations for patients at different risk levels. Clinicians often make treatment decisions directly based on examination results, while risk classification often plays an indirect role. For risk scores to play a more effective role in the management of HF, clinical trials are needed to explore the safety and effectiveness of individualized treatment strategies based on risk assessment, thereby forming a higher level of evidence. It is expected that in the future, the risk score can directly guide clinical medication and treatment in the same way as the atrial fibrillation CHA2DS2-VASc score (92).

## Limitations and strengths

This study has the following strengths: we systematically retrieved all well-performing prediction models for HF prognosis that have undergone external validation since the databases were established and assessed the risk of bias in the included models using the PROBAST tool. Furthermore, the study categorized the retrieved models based on their target populations, encompassing models with HFrEF, HFmrEF, and HFpEF, as well as models for all types of HF, with each category discussed separately. By comparing the similarities and differences among models within the same category and providing a detailed evaluation of their strengths and limitations, this review provides clear guidance for researchers selecting appropriate models. Concurrently, this study summarized the essential characteristics of high-quality prediction models across multiple dimensions, including cohort selection, predictive factor screening, and model presentation formats. The review also outlined the latest advances in current prediction model development, offering valuable reference points for future research.

However, this study also has several limitations. Given the large number of HF prognosis prediction models, to ensure the robustness and clinical applicability of the included models, we excluded those without external validation (typically indicating insufficient evidence of predictive efficacy and generalizability). This approach may have resulted in incomplete coverage of models. Nevertheless, we included models that, although not externally validated during their original development, subsequently underwent external validation in other studies. Furthermore, novel biomarkers and modeling methods reported in studies without external validation were also discussed in our study, thereby partially mitigating this limitation.

## Conclusion

In summary, this systematic review included 38 studies and provided a detailed discussion of 46 prediction models for prognostic risk stratification across different types of HF. Analysis revealed that most models exhibited some degree of bias risk, suggesting that future research should fully adhere to PROBAST criteria when developing models, employing standardized methodologies to construct models with better predictive performance. Furthermore, the latest and most comprehensive strategies should be adopted in aspects such as cohort selection, predictor screening, model construction methods, and presentation formats. This approach will facilitate the development of high-quality prediction models that demonstrate robust predictive performance, strong generalizability, and clinical utility. In addition, prediction models that have consistently demonstrated strong performance through multiple external validations should be incorporated into clinical guidelines to enhance their practical application.
